# The use of photosynthetic pigments and SPAD can help in the selection of bean genotypes under fertilization organic and mineral

**DOI:** 10.1038/s41598-023-49582-4

**Published:** 2023-12-18

**Authors:** José Andres Carreño Siqueira, Douglas José Marques, Maria Clara Gabriel Silva, Cristian Araújo Silva

**Affiliations:** Instituto de Ciências Agrárias, Universidade Federal de Uberlândia (UFU), Rodovia LMG 746, Km 01, Bairro Araras, Bloco 1, Monte Carmelo, Uberlândia, MG Brazil

**Keywords:** Agroecology, Abiotic

## Abstract

The common bean is cultivated in all regions of the country, representing a product of great economic and social importance. In order to ensure food security in the world, it is necessary to create alternatives to reduce the dependence on fertilizers and seeds, and in this context, organic agriculture is a sustainable alternative to ensure it. Therefore, it becomes necessary to adapt rapid methods to monitor plant nutrition in real-time. The reflectance index determined by SPAD and pigment determination can be a sustainable alternative to identify genotypes in different fertilizations (organic × mineral fertilizer). The research hypothesis is to monitor nutritional management through pigment levels and reflectance index in common bean cultivars and their adaptation into different types of fertilization (organic × mineral fertilizer). Therefore, the objective of the research was to evaluate the common bean genotypes of the type carioca, in different fertilizations (organic × mineral fertilizer), and their effects on photosynthetic pigments, and the relationship between SPAD reflectance index and productivity. The experimental design used was a 2 × 7 factorial in randomized blocks with four replications: The first factor was the fertilization (organic × mineral fertilizer)and the second were the 7 genotypes (UFU-1; UFU-2; UFU-3; UFU-4; UFU-5; UFU-6 UFU-7), with UFU-1 being a hybrid obtained between genotypes UFU-4 and UFU-7; UFU-2 and UFU-3 were commercial genotypes; and UFU-4, UFU-5, UFU-6 and UFU-7 were genotypes from the UFU germplasm bank, located in the city of Monte Carmelo, Brazil. Evaluations were carried out for the agronomic characteristics of the plants, which were: height, number of branches, length and volume of roots, dry matter, leaf area index, number of flowers, number of pods, number of seeds per pod, 100 seed weight, and productivity of the genotypes. The results were compared with chlorophyll content and SPAD reflectance index, and the genotypes showed distinct behavior for each fertilization (organic × mineral fertilizer). The genotypes recommended for the organic fertilizer were UFU-2, UFU-6, and UFU-7, which showed higher productivity. For themineral fertilizer, the best-adapted genotype was UFU-4, with a higher productive yield. In conclusion, we can affirm that the highest chlorophyll and SPAD indices can help select common bean genotypes with higher productivity and adaptation within the organic fertilizer being this the main focus of this research. However, the other variables carried out during this research also demonstrated to have significant effects, so they could be analyzed individually and could offer valuable information in the selection of the best-adapted genotypes.

## Introduction

Food security refers to the availability of food and population's access to it. Beans have a nutritional value that is considered suitable to help reduce malnutrition in the population of developing countries^1^. Food consumption largely depends on consumers' financial conditions, and beans hold a prominent place due to their low cost and high nutritional value. In this context, the cultivation of beans within the organic fertilizer would provide a healthy food source, as well as a sustainable cultivation model^2^.

Brazil has a prominent position in the cultivation of this legume, being both a producer and consumer, contributing with about 25% of the protein consumed by the low-income population. Its cultivation is practiced by subsistence farmers, with low application of production technologies^3^, mainly associated with family farming and small farmers.

Within the focus of organic agriculture, one of the main objectives is the development of sustainability. Therefore, due to the input restrictions within the organic fertilizer**s**^1^, the cultivation of organic beans must adopt the use of renewable inputs^4^. Hence, the improvement of traditional crop management techniques, as well as the use of local biodiversity, become necessary to provide a healthy and sustainable food source for consumers^5^.

The organic sector has developed greatly in recent years, despite facing the problem of low availability of seeds adapted to this type of fertilization, as they should preferably come from organic fertilizers^1^.

Achieving the maximum potential of a crop through production management represents a significant challenge. Consequently, to facilitate this process, the quantification of chlorophyll content and SPAD index has been shown as viable alternatives^6^. These methods facilitate the process of identifying better-adapted bean genotypes^7^, given that a positive correlation has been found between the SPAD index and productivity, as leaves with the highest chlorophyll content tend to be associated with higher productivity^8,9^.

The determination of chlorophyll reflectance has become an important technique for studying plant physiology and ecology^10^, because the total chlorophyll content per leaf area can be quickly and non-destructively estimated using a portable SPAD chlorophyll meter. SPAD readings are calculated based on two transmission values: red light at 650 nm, which is absorbed by chlorophyll, and infrared light at 940 nm, which is not absorbed by chlorophy^9^.

One of the major challenges in the production of organic beans today is the lack of cultivars adapted to the use of organics fertilizers. There is a wide variety of suitable material available, but it is dispersed in the form of local and wild cultivars, which is why it is necessary to identify the material that is best adapted to the organic fertilizers^5^.

Bean productivity in Brazil is considered low^11^, which may be attributed to the low level of technology adopted by small farmers. Therefore, it is necessary to identify genotypes with high productivity stability in different fertilizations (organic × mineral fertilizer), taking into account agronomic performance, nutritional and commercial quality of the grains, for good acceptance by producers and consumers^12^.

The growing concern for healthy eating has led to an increase in demand for products from these systems, leading in turn to the development of agroecological systems. Therefore, the organic fertilizers would need adaptation to improve soil fertility management, as well as the adoption of cultivars that respond better to fertilizers used in this systems^13^.

Thus, the research aimed to evaluate common bean genotypes, carioca type, in different fertilizers (organic × mineral fertilizer), and their effects on photosynthetic pigments, and the relationship between the SPAD reflectance index and productivity.

## Results

### The genotypes and fertilizers (organic × mineral fertilizer)

**Figure 1:**
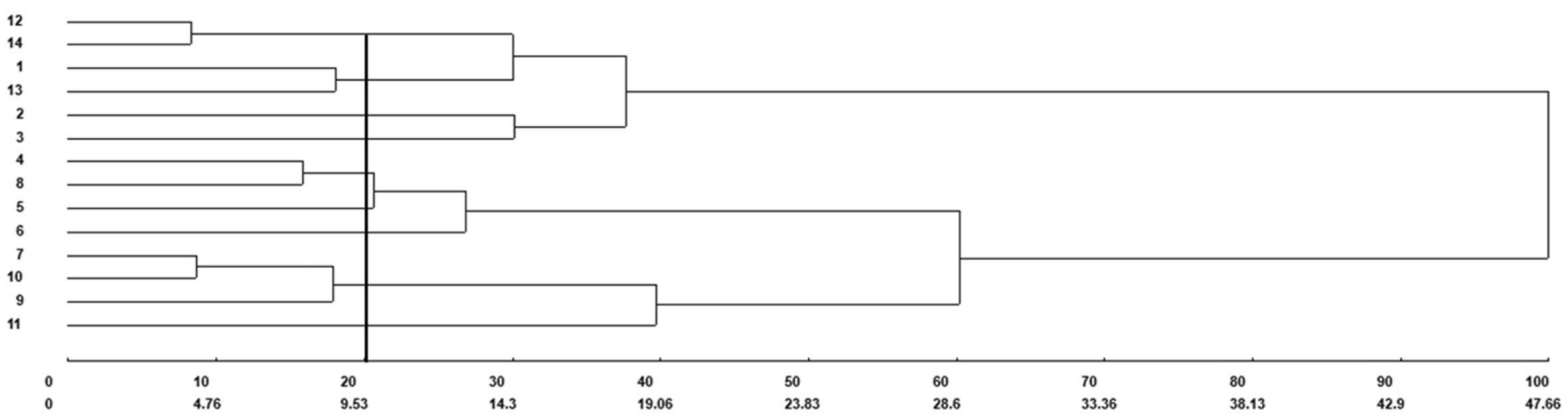
Illustrative dendrogram of the analysis of fourteen treatments by the unweighted pair group method with arithmetic mean (UPGMA) obtained with Mahalanobis distance, with the mean generated from 10 variables according to the genotypes and different fertilization (organic × mineral fertilizer). Common bean genotypes grown in the organic fertilizer (1 = UFU-1; 2 = UFU-2; 3 = UFU-3; 4 = UFU-4; 5 = UFU-5; 6 = UFU-6; 7 = UFU-7) and genotypes grown in the mineral fertilizer (8 = UFU-1; 9 = UFU-2; 10 = UFU-3; 11 = UFU-4; 12 = UFU-5; 13 = UFU-6; 14 = UFU-7) for common bean plants.

The analysis of the fourteen treatments was carried out using the unweighted pair group method with arithmetic mean (UPGMA) with Mahalanobis distance, with the mean generated from 10 variables according to the genotypes and fertilization (organic × mineral fertilizer). Common bean genotypes grown under organic fertilizer (1 = UFU-1; 2 = UFU-2; 3 = UFU-3; 4 = UFU-4; 5 = UFU-5; 6 = UFU-6; 7 = UFU-7) and genotypes under mineral fertilizer(8 = UFU-1; 9 = UFU-2; 10 = UFU-3; 11 = UFU-4; 12 = UFU-5; 13 = UFU-6; 14 = UFU-7) for common bean plants (Fig. 1).

The grouping was carried out from a cutoff line considering 20% similarity among treatments. The cutoff line was established where an abrupt change was observed in the branches present in the dendrogram^14^. The hierarchical UPGMA method formed groups with a cophenetic correlation coefficient of 72%, making it possible to affirm that the dendrogram satisfactorily reproduced the information contained in the matrix and, consequently, in the formation of groups (Table 1). The greatest relative contribution was for total chlorophyll.

**Table 1:** Relative contribution (%) of characteristics to dissimilarity, estimated by the method proposed by^15^ according to the genotypes and fertilizer (organic × mineral fertilizer).

Characteristics for dissimilarity	Relative contribution (%)
Total chlorophyll	47.63
Leaf area index (cm^2^)	2.19
100-seed weight s (g)	14.22
Total dry mass	6.62
Productivity (g plant^−1^)	6.98
Main stem	2.38
Secondary stem	6.40
Reproductive branches	9.21
Flowers	0.58
Flowering index	4.16
Total	100.00

### Root genotypes common beans

**Figure 2:**
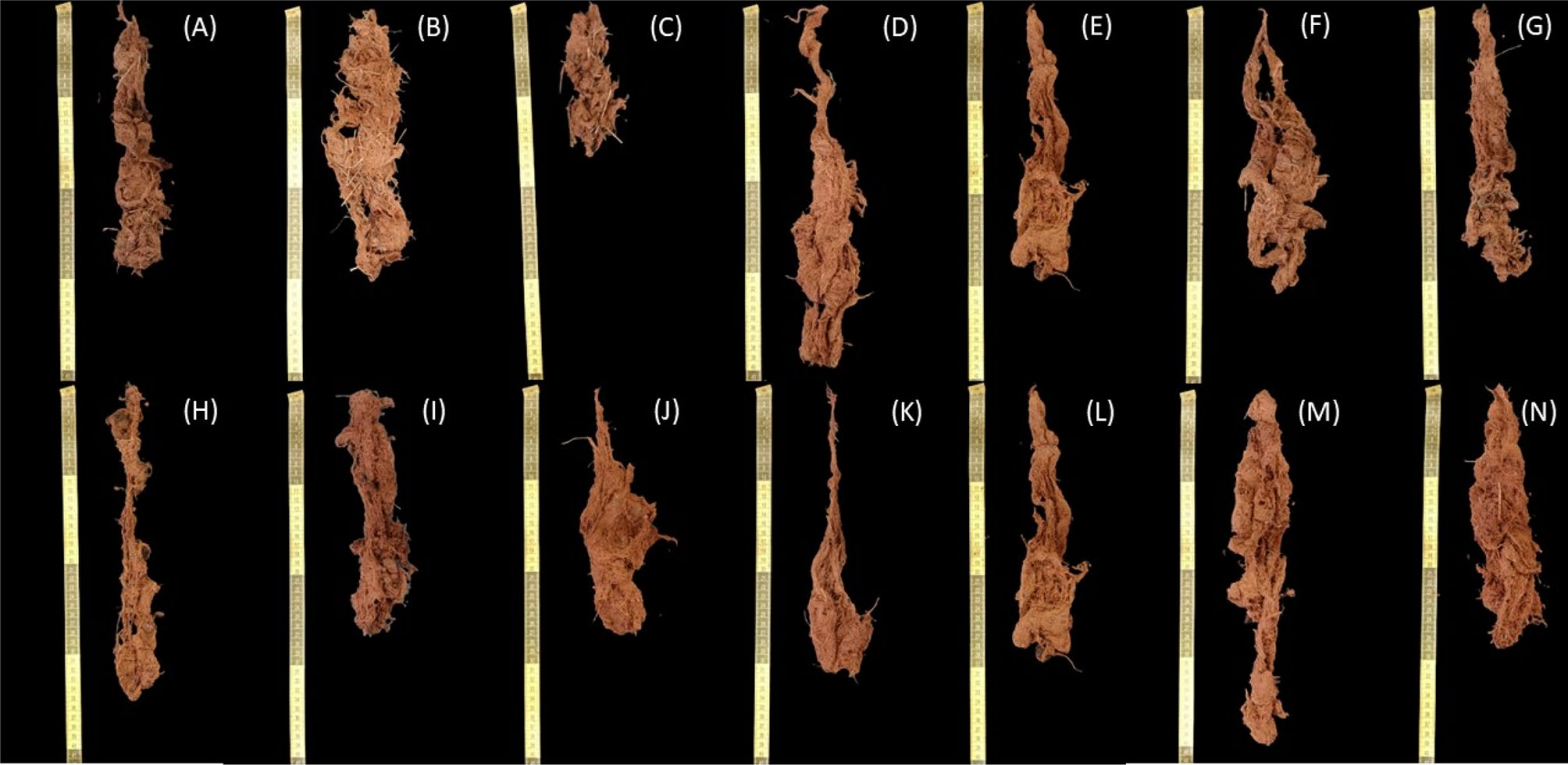
Root of common bean genotypes. UFU-1 (**A**); UFU-2 (**B**), UFU-3 (**C**), UFU-4 (**D**); UFU-5 (**E**), UFU-6 (**F**), and UFU-7 (**G**) under organic fertilizer and UFU-1 (**H**); UFU-2 (**I**), UFU-3 (**J**), UFU-4 (**K**); UFU-5 (**L**), UFU-6 (**M**), and UFU-7 (**N**) under mineral fertilizer.

Figure 2 shows the root of common bean cultivars under fertilization (organic × mineral fertilizer).

**Figure 3:**
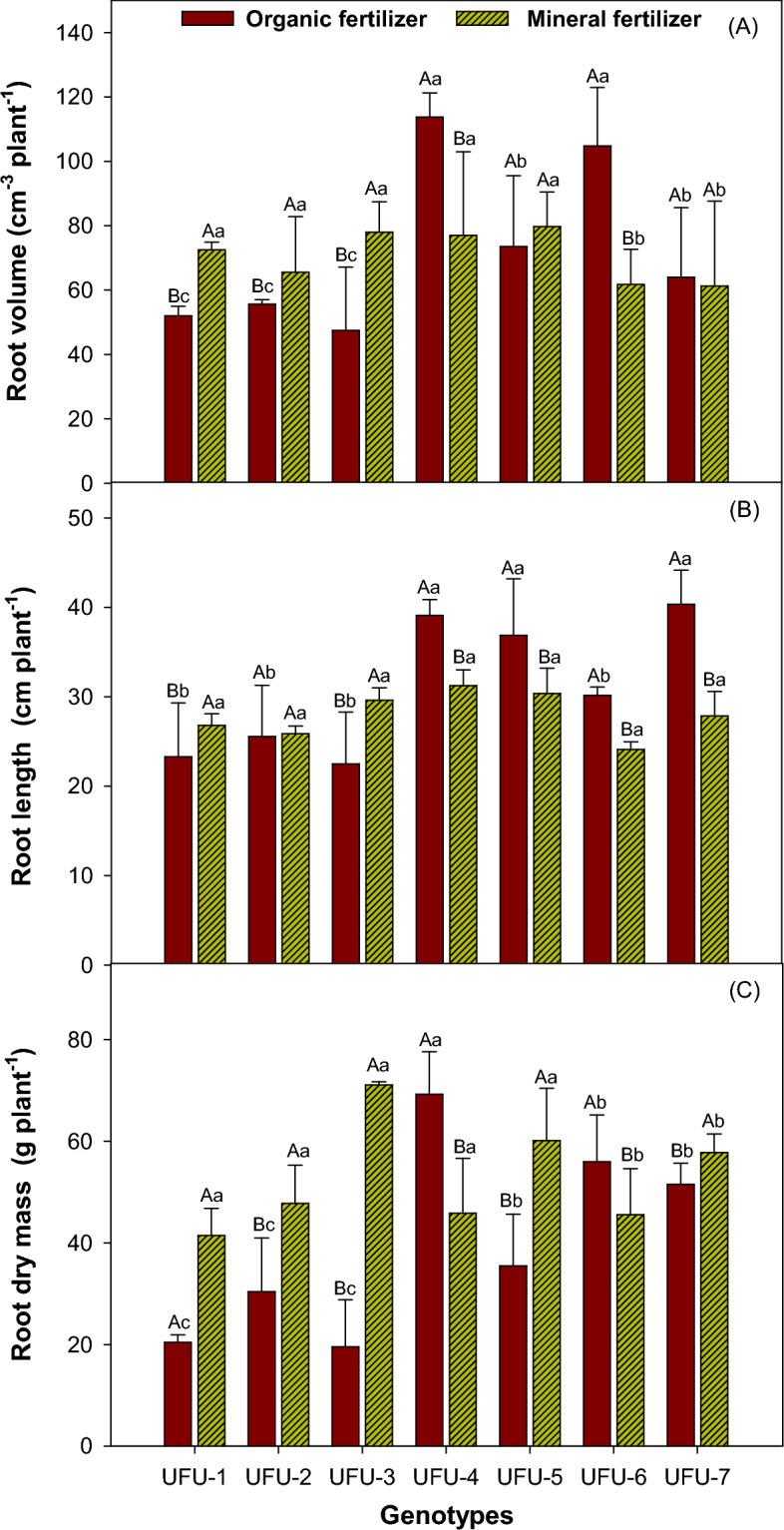
Root volume (**A**), root length (**B**), and root dry mass (**C**) as a function of genotypes and in different fertilization (organic × mineral fertilizer). Columns with different uppercase letters (different colors) compare different fertilization (organic × mineral fertilizer), and lowercase letters (same color) compare genotypes, indicating significant differences from the Scott-Knott test (*P* < 05). Columns correspond to means of four repetitions and standard deviations.

For the interaction between fertilization, the UFU-4 (32.31%) and UFU-6 (41.06%) genotypes presented a greater root volume for organic fertilization. For comparison within the mineral fertilizer, UFU-6 (41.05%) and 7 (3.51%) produced the smallest root volumes (Fig. 3A). For root length, the UFU-4 (20.07%), 5 (17.62%), and 7 (30.90%) genotypes were superior for the interaction between fertilizer (Fig. 3B). Finally, regarding root mass, within the interaction, in the organic fertilizer, the UFU-4 (33.79%) and 6 (18.63%) genotypes presented higher values, and for the mineral fertilizer were the UFU-3 (72.46%), 5 (33.79%), and 7 (10.80%) genotypes (Fig. 3C).

### Genotypes common beans

**Figure 4:**
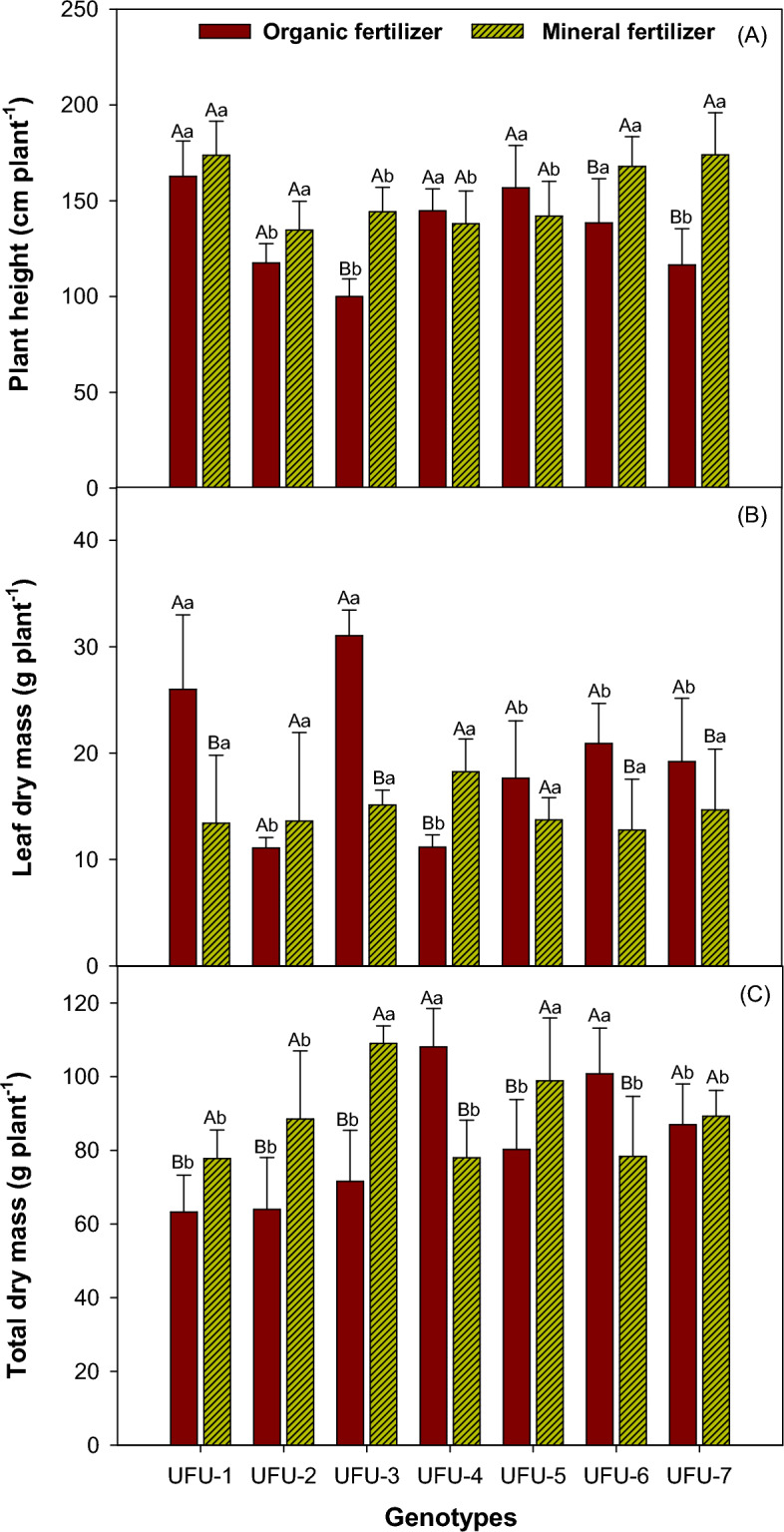
Plant height (**A**), leaf dry mass (**B**), and total dry mass (root + stem + leaf) (**C**) as a function of genotypes and in different fertilization (organic × mineral fertilizer). Columns with different uppercase letters (different colors) compare different fertilization (organic × mineral fertilizer) and lowercase letters (same color) compare genotypes indicating significant differences from the Scott-Knott test (*P* < 05). Columns correspond to means of four replicates and standard deviations.

For bean plant height in the fertilization (organic × mineral fertilizer) in the interaction (Fig. 4A), the UFU-1 (6.33%), UFU-6 (17.55%), and UFU-7 (33.04%) genotypes presented 170 cm in height within the mineral fertilizer. In the organic fertilizer, the plants with the lowest height were UFU-2 (12.80%), UFU-3 (30.67%), and UFU-7 (33.04%). For leaf dry mass (Fig. 4B), the UFU-1 and UFU-3 genotypes presented the highest leaf mass volume, between 25 and 30 g in organic fertilization, 41.68% and 51.73% higher respectively compared to the previous ones. For total dry matter (root + stem + leaf), (Fig. 4C) for mineral fertilizer, the UFU-3 (34.31%) and UFU-5 (18.83%) genotypes presented the highest mass values between 110 and 100 g, respectively. For the organic fertilizer, the UFU-4 (27.82%) and UFU-6 (22.22%) genotypes presented the highest volumes of common bean plant mass (Fig. 4C). Comparing mineral fertilizer and organic fertilizer cultivation for plant height, the UFU-6 and UFU-7 genotypes were the only ones that presented a significant difference, with higher values in the mineral fertilizer.

### Leaf area index common beans

**Figure 5:**
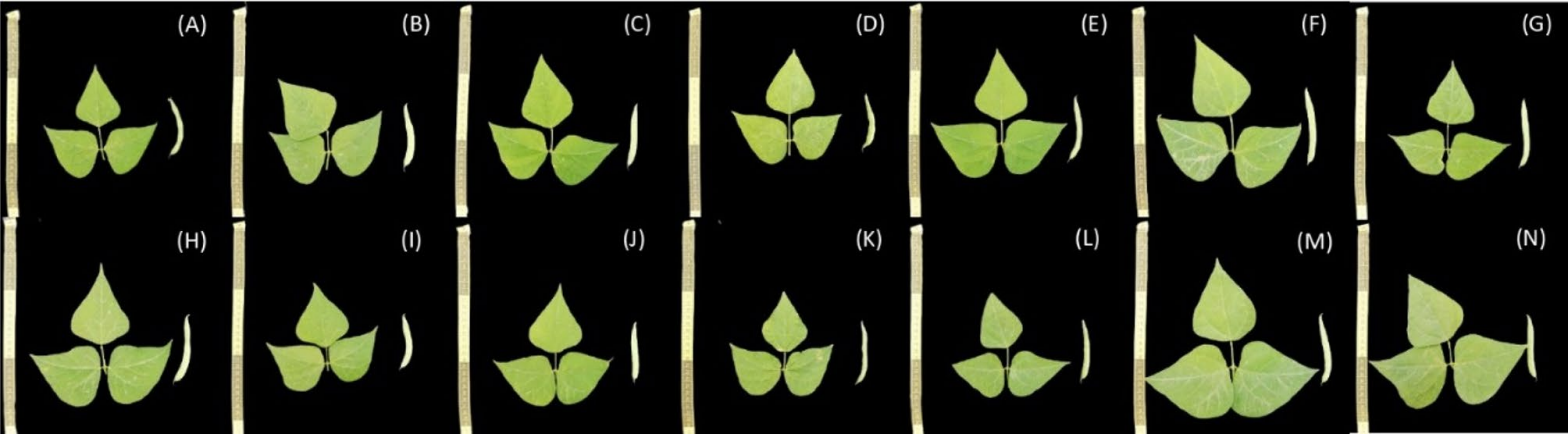
Leaves and pods of common bean genotypes UFU-1 (**A**); UFU-2 (**B**), UFU-3 (**C**), UFU-4 (**D**); UFU-5 (**E**), UFU-6 (**F**) and UFU-7 (**G**) under organic fertilizer and genotypes UFU-1 (**H**); UFU-2 (**I**), UFU-3 (**J**), UFU-4 (**K**); UFU-5 (**L**), UFU-6 (**M**) and UFU-7 (**N**) under mineral fertilizer.

Figure 5 shows the leaves and the green vegetable of each of the genotypes planted in the experiment, demonstrating little variation between fertilization (organic × mineral fertilizer) as well as between them, therefore the differences found in the experiment are due to greater production of leaves rather than a variation in their size.

**Figure 6:**
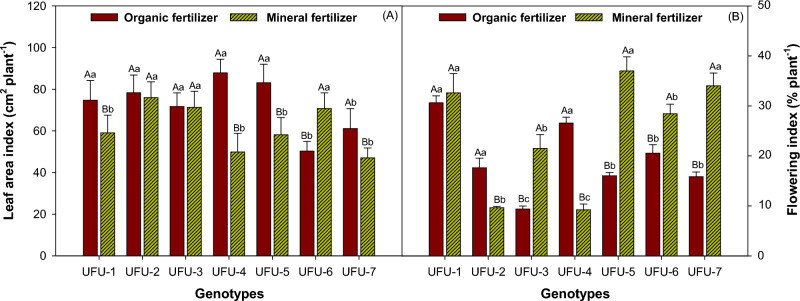
Leaf area index (**A**) and flowering (**B**) as a function of genotypes and in different fertilization (organic × mineral fertilizer). Columns with different uppercase letters (different colors) compare different fertilization (organic × mineral fertilizer), and lowercase letters (same color) compare genotypes indicating significant differences from the Scott-Knott test (*P* < 05). Columns correspond to means of four repetitions and standard deviations.

The highest leaf area index (Fig. 6A) was achieved by genotypes UFU-1 (20.93%), UFU-2 (3%), UFU-3 (0.44%), UFU-4 (43.25%), and UFU-5 (30.06%) under the organic fertilizer. In the mineral fertilizer, genotypes UFU-2, UFU-3, and UFU-6 (28.89%) were superior. The genotypes with the highest leaf area index occurred in the organic fertilizer. Regarding the flowering index (Fig. 6B), under the organic fertilizer, genotypes UFU-1 and UFU-4 (65.28%) were superior, while under the mineral fertilizer, genotypes UFU-1 (6.09%), UFU-5 (56.67%), and UFU-7 (53.43%) presented considerably higher results.

### Bean leaf chlorophyll and SPAD

**Figure 7:**
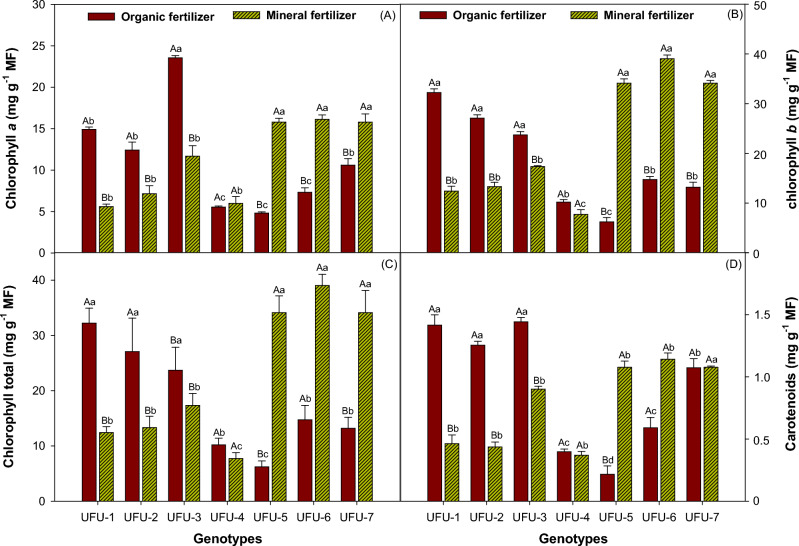
Chlorophyll *a* (**A**), chlorophyll *b* (**B**), total chlorophylls (**C**), and carotenoids (**D**) as a function of genotypes and in different fertilization (organic × mineral fertilizer). Columns with different uppercase letters (different colors) compare different fertilization (organic × mineral fertilizer) and lowercase letters (same color) compare genotypes and indicate significant differences from the Scott-Knott test (*P* < 05). Columns correspond to means of four repetitions and standard deviations.

For chlorophyll *a*, chlorophyll *b*, total chlorophyll, and carotenoid content (Fig. 7), genotypes UFU-1, UFU-2, and UFU-3 in the organic fertilizer reached levels that were 35.74%, 42.06%, and 31.36%. Genotypes UFU-5, UFU-6, and UFU-7 were superior in pigment production in the mineral fertilizer 16.25%, 46.22%, and 56.93% higher than the organic fertilizer. For chlorophyll *a* content, genotypes UFU-1, UFU-2, and UFU-3 showed levels 37.55%, 57.64%, and 49.63% higher within the organic fertilizer, and genotypes UFU-5, UFU-6, and UFU-7 showed levels 30.44%, 45.44%, and 67.02% higher within the mineral fertilizer. Chlorophyll *b* levels were 39.34%, 41.99%, and 28.84% higher for UFU-1, UFU-2, and UFU-3, respectively, in the organic fertilizer, while genotypes UFU-5, UFU-6, and UFU-7 showed levels 36.71%, 86.55%, and 40.28% higher, respectively, within the mineral fertilizer. For carotenoids (C) as a function of genotypes and fertilization (organic × mineral fertilizer), UFU-1, UFU-2, and UFU-3 genotypes showed higher levels within the organic fertilizer (32.39%, 35.2%, and 62.2%, respectively). Genotypes UFU-5, UFU-6, and UFU-7 had higher carotenoid levels within the mineral fertilizer, which showed similar values among them.

**Figure 8:**
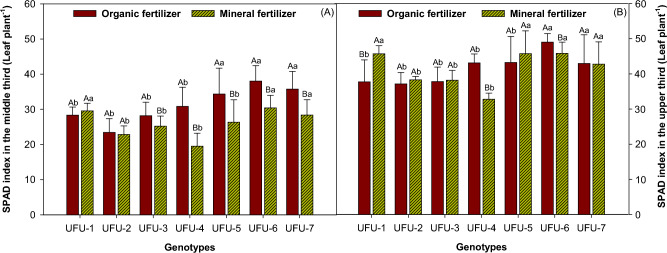
SPAD index in the middle third of the common bean plant (**A**) and SPAD index in the upper third (**B**) of the plant as a function of genotypes and in different fertilization (organic × mineral fertilizer). Columns with different uppercase letters (different colors) compare different fertilization (organic × mineral fertilizer), and lowercase letters (same color) compare genotypes, indicating significant differences from the Scott-Knott test (*P* < 0.05). Columns correspond to means of four repetitions and standard deviations.

The SPAD index was measured in the middle third of the plant (Fig. 8A), which showed a notable superiority for organic fertilization, as reflected in genotypes UFU-3, UFU-4, UFU-5, UFU-6, and UFU-7, presenting an index of 10.58%, 36.66%, 23.35%, 20.07%, and 20.62% higher than the mineral fertilizer. For the SPAD index measured in the upper third of the plant (Fig. 8B), the results were statistically similar for genotypes UFU-2, UFU-3, and UFU-5 for both fertilizations (organic × mineral fertilizer). Genotype UFU-1 (17.44%) was superior in the mineral fertilizer when compared to the organic fertilizer, where genotypes UFU-4 (23.97%), UFU-6 (6.50%), and UFU-7 (0.30%) showed superior results.

### Ramifications in the common beans plant

**Figure 9:**
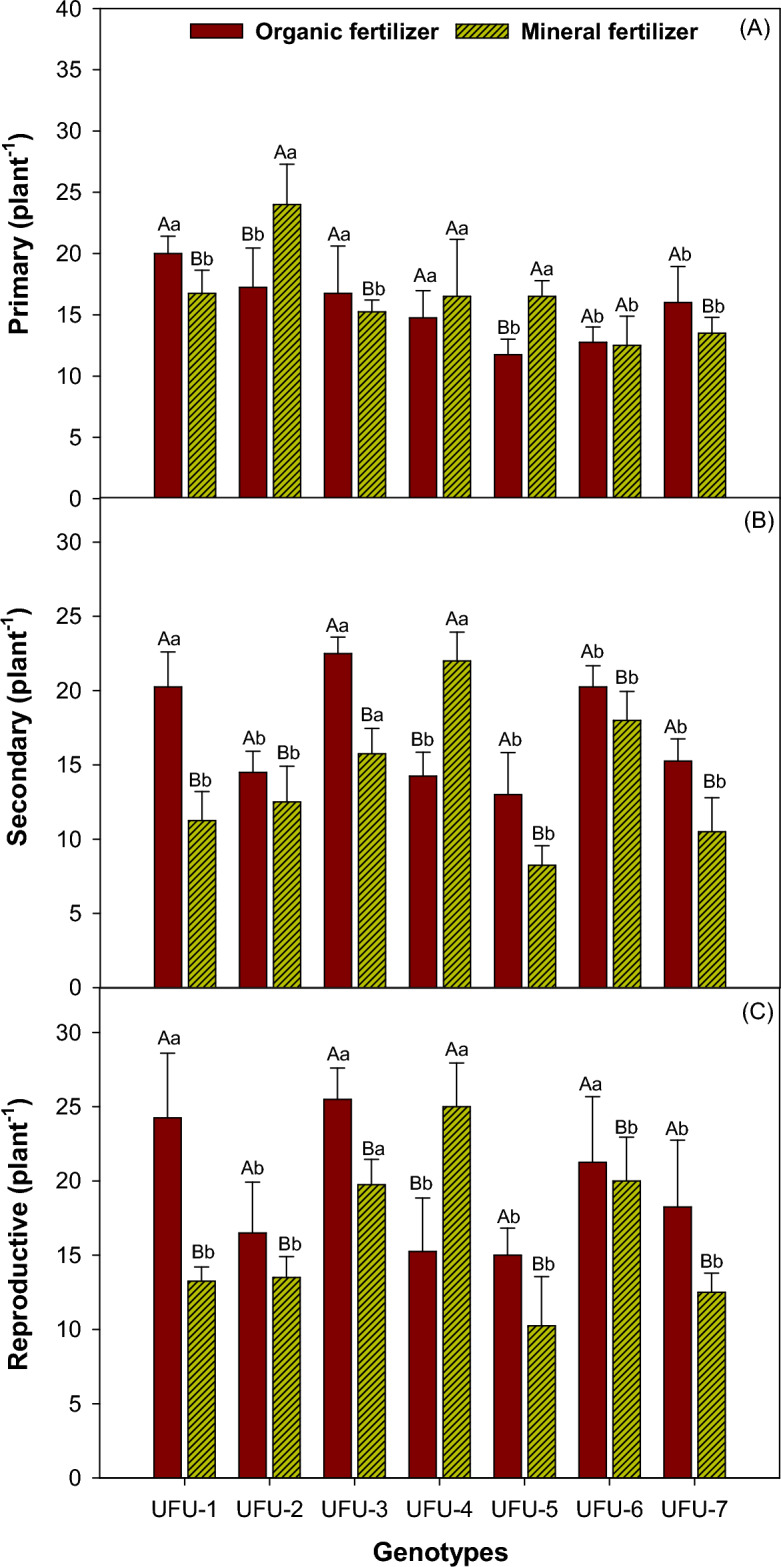
Primary (**A**), secondary (**B**), and reproductive (**C**) branches of common bean as a function of genotypes and in different fertilization (organic × mineral fertilizer). Columns with different capital letters (different colors) compare different fertilization (organic × mineral fertilizer), and lowercase letters (same color) compare genotypes indicating significant differences from the Scott-Knott test (*P* < 05). Columns correspond to the means of four repetitions and standard deviations.

Mineral fertilizerproved to be notably superior in terms of the number of primary branches (Fig. 9A) for genotypes UFU-2 (28.12%) and UFU-5 (28.78%). Genotypes UFU-1 (16.25%), UFU-3 (8.95%), and UFU-7 (15.62%) were superior within the organic fertilizer, and genotypes UFU-4 and UFU-6 showed statistically similar results for both types of fertilization. For the number of secondary branches (Fig. 9B), genotypes UFU-1 (45.36%), UFU-2 (18.18%), UFU-3 (22.54%), UFU-5 (31.66%), UFU-6 (5.88%), and UFU-7 (31.50%) were superior in organic fertilization. Genotype UFU-4 was significant superior for the mineral fertilizer and genotype UFU-3 was significant superior for the organic fertilizer. For the number of reproductive branches (Fig. 9C), genotypes UFU-1 (31.63%), UFU-2 (5.60%), UFU-3 (8.24%), UFU-5 (25%), UFU-6 (10.84%), and UFU-7 (3.65%) were superior within organic fertilization. The most significant result was for genotype UFU-3 (21.78%), which showed superiority in the mineral al fertilizers..

### Productivity of common beans

**Figure 10:**
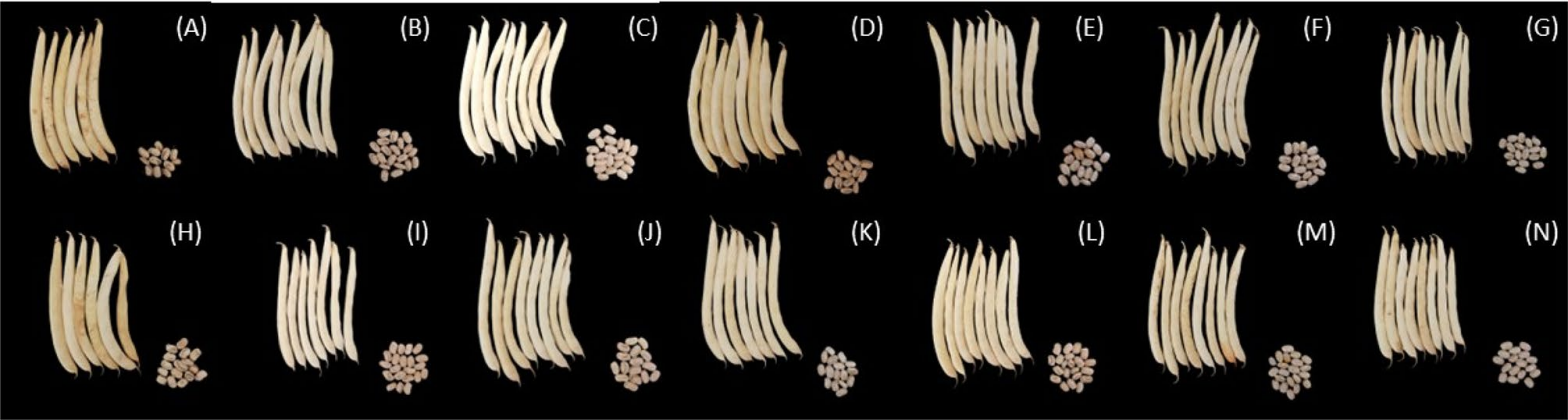
Pods and grains of common bean in UFU-1 (**A**); UFU-2 (**B**), UFU-3 (**C**), UFU-4 (**D**); UFU-5 (**E**), UFU-6 (**F**) and UFU-7 (**G**) under organic fertilizer and UFU-1 (**H**); UFU-2 (**I**), UFU-3 (**J**), UFU-4 (**K**); UFU-5 (**L**), UFU-6 (**M**), and UFU-7 (**N**) under mineral fertilizer.

In Fig. 10, the vegetables after harvesting can be seen, as well as their seeds, which did not show significant differences in the agronomic or descriptive characteristics, demonstrating that the cultivars chosen for the present experiment have very similar characteristics.

**Figure 11:**
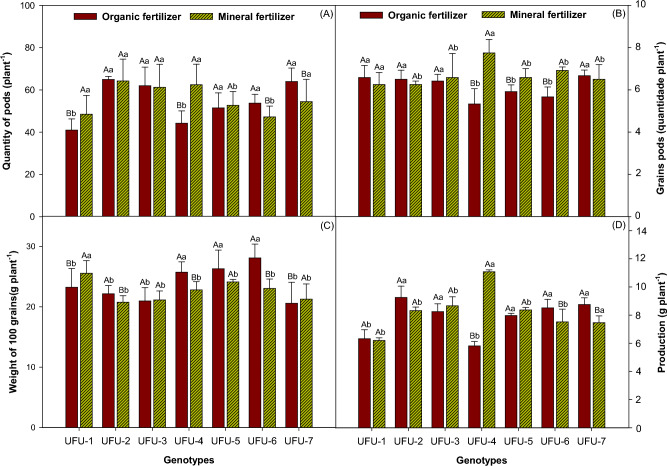
Quantity of pods (**A**), grains (**B**), weight of 100 grains (**C**), and productivity (**D**) of common bean as a function of genotypes and in different fertilization (organic × mineral fertilizer). Columns with different uppercase letters (different colors) compare different fertilization (organic × mineral fertilizer), and lowercase letters (same color) compare the genotypes, indicating significant differences from the Scott-Knott test (*P* < 05). Columns correspond to means of four repetitions and standard deviations.

The highest number of pods (Fig. 11A) was found in genotypes UFU-2, UFU-3, and UFU-5, which remained similar for both fertilization (organic × mineral fertilizer). Genotypes UFU-1 (15.46%) and UFU-4 (29.2%) were superior in the mineral fertilizer compared to genotypes UFU-6 (12.09%) and UFU-7 (14.84%), which were superior in the organic fertilizer. For the number of seeds per pod (Fig. 11B), there was no significant difference in fertilization (organic × mineral fertilizer) for genotypes UFU-1, UFU-2, UFU-3, and UFU-7. However, genotypes UFU-4 (26.48%), UFU-5 (11.38%), and UFU-6 (18.03%) were superior for mineral fertilizer. However, for the weight of 100 grains (Fig. 11C), genotypes UFU-2 (6.31%), UFU-4 (11.38%), UFU-5 (8.28%), and UFU-6 (18.03%) in the organic fertilizer were superior. On the other hand, genotypes UFU-1 (9.07%) and UFU-7 (3.24%) were superior in the mineral fertilizer. Genotype UFU-3 showed good results in both fertilization (organic × mineral fertilizer).

The productivity of the plants measured in grams per plant (Fig. 11D) remained similar for the UFU-1 and UFU-5 genotypes, for the UFU-2, UFU-6 and UFU-7 genotypes they were superior within the organic fertilizer, this being 10%, 11% and 14% respectively, the UFU-3 genotype was slightly superior in the mineral fertilizer (4%), the UFU-4 genotype was notably superior in the mineral fertilizer (47%).

**Figure 12:**
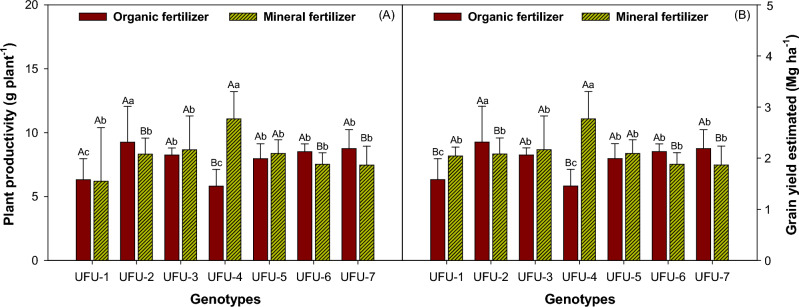
Shows the plant productivity (g plant^−1^) (**A**) and grain yield estimated (Mg ha^−1^) (**B**) of common bean as a function of genotypes and in different fertilization (organic × mineral fertilizer). Columns with different uppercase letters (different colors) compare different fertilization (organic × mineral fertilizer), and lowercase letters (same color) compare the genotypes, indicating significant differences from the Scott-Knott test (*P* < 05). Columns correspond to means of four repetitions and standard deviations.

For comparative reasons it was considered in linear meter considering 10 plants per meter, with 0.4 m spacing between plants (Fig. 12A) and graind yield per hectare (Fig. 12B), genotype UFU-4 showed higher productivity in the mineral fertilizer . However, for the organic production fertilizer, the highest productivity was for genotype UFU-2 (Fig. 12A,B).

Regarding plant productivity (Fig. 12A), genotypes UFU-1, UFU-3 e UFU-5 were statistically similar for both fertilizers (organic × mineral fertilizer), while genotype UFU-4 showed the higest yield in the mineral fertilizer 47.47% superior.

## Discussion

Fertilizers of organic and mineral origin have been widely compared, so normally, due to the fact that organic fertilizer has a slower release of nutrients, they tend to have a certain disadvantage when compared to readily available fertilizers of mineral origin^26^, which can be observed in a greater accumulation of dry matter in the aerial part, as well as in the other evaluations carried out in the present experiment^39^. On the other hand, organic matter improves the physical–chemical characteristics of the soil, which may explain the greater development of the root system in some cultivars^37^.

In the present study, it was observed that organic fertilizer was beneficial in some physiological aspects of the plant, such as leaf area index (LAI) and number of branches, which usually positively affect plant yield. The efficiency of fertilization in chlorophyll production was not reflected in higher productivity, which could be attributed to low photosynthetic efficiency, as well as adverse effects at high temperatures during the course of this work^36^. This phenomenon can be explained due to the fact that it has been demonstrated that the use of organic fertilizers tends to mitigate the effects caused by stress on plants, as well as that organic matter has proven to promote the production of some hormones such as auxin, in addition, organic matter has shown to be a constant source of nitrogen release, which is much better available due to its slower release when purchased at the mineral allowance, which is an essential macronutrient for plant growth^21,22^.The LAI values were mostly higher for organic fertilization, which agrees with several studies that have shown that the use of organic compounds increases LAI when compared to unfertilized controls^29,37^.

When comparing the productive yield of fertilization (organic × mineral fertilizer), there is generally a notable superiority for chemical fertilization^38^, but on the other hand, the organic fertilizer has shown a higher LAI when compared to other polymers used in the market^39^. Experiments carried out with cultivars showed a higher LAI under mineral fertilizer, but some cultivars showed a higher index within the organic fertilizer, thus demonstrating the existence of cultivars better adapted to the organic material as fertilizer^40^. However, the different types of organic fertilization tested in the experiment tended to show a higher leaf area index compared to the mineral fertilization^41^.

Maintaining chlorophyll levels is important for flower preservation since their decrease, due to adverse effects of fertilization (organic × mineral fertilizer), can cause flower abortion, thereby decreasing the final crop yield^42,43^. Since leaves are the main productive unit of the plant and stimulate a higher photosynthetic rate^52^ these results were observed by the positive relationship between LAI and the number of pods^44^.

Two methodologies were used to evaluate chlorophyll levels in the present experiment. The first was performed in the laboratory using a spectrophotometer to observe chlorophyll indices (*a, b*, and total) in different tested genotypes, while the second was performed using a portable chlorophyll meter (SPAD-502), which provides instant non-destructive readings.

A significant interaction between plant fertilization and chlorophyll levels was observed^16^, which may indicate a positive interaction between the fertilizer and the plant. In addition, an increase in chlorophyll levels was also observed for the application of organic fertilizer in the form of sewage sludge^17^.

The results of the present experiment are consistent with those found in the literature, where an increase in carotenoid levels was observed in plants fertilized with organic compost^18^. However, a decrease in carotenoid levels was also observed in the application of sewage sludge^19^, as well as a lack of variation in carotenoid levels in different fertilization (organic × mineral fertilizer)^20^. Therefore, it can be concluded that chlorophyll levels depend on the nutrient levels provided to the plant, and there is a wide variety of organic materials that can be used.

The results obtained in the present experiment regarding chlorophyll content can be explained by the fact that there is a direct link to fertilization, specifically to nitrogen, as it is an essential nutrient in the structure of the molecule of different types of chlorophyll. The high influence of nitrogen fertilization is due to the fact that 50% to 70% of the total N in leaves is associated with chloroplasts, and under conditions of nutrient deficiency, the plant tends to translocate N to regions of active growth^21^.

Chlorophyll is the essential pigment for photosynthesis. It becomes excited when it absorbs light, reaching a higher energy state and becoming unstable, releasing energy in the form of heat, which is captured, allowing photosynthetic processes to occur^22^.

The SPAD index also allows for the calculation of chlorophyll content, as well as being a method used to estimate the amount of nitrogen assimilated by plants^23^. The SPAD reflectance index can be used as an estimate to determine crop productivity^24^ and the results obtained correlated with productivity in the bean crop. These results are in agreement with those presented^25^, where an increase in chlorophyll content was observed in plants treated with organic compost. But on the other hand in the comparison between fertilization (organic × mineral fertilizer), the genotypes UFU-1, UFU-2 and UFU-3 showed higher levels of chlorophyll under mineral fertilizer meanwhile the genotypes UFU-5, UFU-6 and UFU-7 showed showed higher levels of chlorophyll under the organic fertilizer, the genotype UFU-4 showed no significant differences between the two fertilizers and showed also the lowest levels of cholophyll^26,27^.

As previously mentioned, adverse effects can negatively alter chlorophyll levels, with the highest levels being obtained in non-stressed treatments, which is beneficial for vegetative growth and greater chlorophyll production, with better-adapted genotypes having higher chlorophyll levels^28^. Although pod yield and quality are related to genetic factors, a direct correlation has been shown between chlorophyll levels and bean productivity, with variability in SPAD levels observed depending on the cultivars studied^52^.

Therefore, chlorophyll concentration is dependent on proper plant nutrition, with organic fertilization tending to have lower chlorophyll levels compared to mineral fertilizer^29^, possibly due to slower availability of organic matter, which directly affects yield.

When comparing yield between organic and mineral, mineral fertilizer tend to have better yields and higher quality pods and seeds^30,31^. Similar results can be observed in corn^32^ and tomato^33^ cultivation. However, yield can vary considerably depending on the techniques used within the organic fertilizer^34^. When comparing creole and conventional varieties under fertilization (organic × mineral fertilizer), commercial varieties tend to have higher productivity, as well as a higher weight of 100 seeds, and better quality, such as seed brightness, which is in agreement with previous studies^35^.

Measuring chlorophyll levels is of great utility for modern agriculture, as it allows for the identification of possible nutritional deficiencies in plants^45^, which results in a valuable ally when evaluating the efficiency of different fertilization (organic × mineral fertilizer)^46^, since it has been shown that an increase in plant chlorophyll levels^29^ translates into greater biomass production^52^.

The total number of pods in the present research, within the organic fertilizer, was similar to those found in the literature with different fertilizer dosages, as was the number of grains per plant^47^. The number of pods obtained in the present experiment was considerably higher than those obtained in the literature^48^ (1.612.00–1.880 Mg ha^−1^). The productivity is within the standard obtained by mineral means (666.70–2571.90 Mg ha^−1^)^61^, and that obtained in the organic fertilizer (1607.00 Mg ha^−1^)^11^. While common bean grown under ideal conditions tends to have a yield of 1.160.70 Mg ha^−1^, the average productivity in the state of Minas Gerais, Brazil, is 1.342.40 Mgha^−1^^11^.

The genotypes that presented the highest SPAD and chlorophyll Indices may be related to higher yields in bean fertilization (organic × mineral fertilizer).

In conclusion, we can affirm that the highest chlorophyll and SPAD indices can help select common bean genotypes with higher productivity and adaptation within the organic fertilizer being this the main focus of this research. However, the other variables carried out during this research also demonstrated to have significant effects, so they could be analyzed individually and could offer valuable information in the selection of the best-adapted genotypes.

## Materials and methods

### Study location and growth conditions

The experiment was conducted in a greenhouse of the arched type, covered with 150-micron plastic film with an additive and the sides covered with white mesh of the anti-aphid type. The research was carried out in a greenhouse located at the Demonstrative and Experimental Field—CaDEx, of the Federal University of Uberlândia, Monte Carmelo-MG Campus (18° 43′ 36.26″ S; 47° 31′ 28.50″ W; 903 m).

The experiment’s first phase began in September 2021, with the sowing of the genotypes and obtaining the hybrid, ending with the harvest in January 2022. The second phase began in August 2022, with the sowing of the genotypes, and concluded in December 2022 with the harvest, agricultural year 2021/2022.

**Figure 13:**
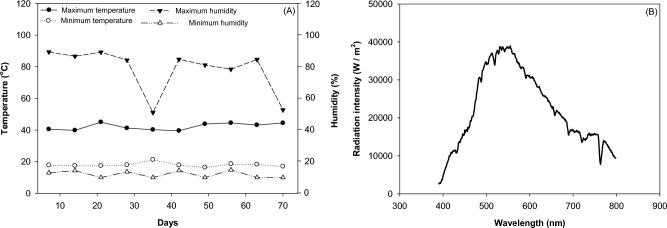
Temperature and humidity (**A**) and radiation intensity (W / m^2^) (**B**) in the greenhouse during the experiment with common bean genotypes in different fertilizations (organic × mineral fertilizer).

The maximum temperature recorded during the research was 42 °C, minimum temperature of 17 °C, maximum humidity of 98%, and minimum of 37.28% (Fig. 13A). The quality and quantity of incident radiation (Luzchem Spectroradiometer, SPR-4002, Ottawa, Canada) for one hour, where the minimum radiation was measured between 390 and the maximum at 790 nm (Fig. 13B).

For organic fertilization, disease, and pest control was carried out using biological control trichoderma, baculovirus, thuringiensis, and aqueous extract of Nim leaves when necessary. For mineral fertilization, disease, and pest control was carried out with spiromesiphene, deltamethrin, avermectins, metalaxyl, and mancozeb.

Irrigation was carried out manually using a millimeter cup, and the amount of water was standardized for all treatments, regulated according to the need, carried out at the field capacity of the pots, and adjusted according to the phenological stage of the plant and the temperature of the location.

### Experimental design

The experimental design used was a 2 × 7 factorial randomized block design, with four replications: the first factor was the fertilization (organic × mineral fertilizer), and the second factor was 7 genotypes of carioca-type common bean (UFU-1; UFU-2; UFU-3; UFU-4; UFU-5; UFU-6 UFU-7). UFU-1 was obtained from the crossing of genotypes (UFU-4 × UFU-7), while two commercial genotypes were also used in the experiment, named UFU-2 and UFU-3, and the accessions from the germplasm bank of the Federal University of Monte Carmelo selected for the experiment were designated UFU-4, UFU-5, UFU-6, and UFU-7. Plastic pots containing 10 dm^3^ kg of soil were used. The applications of different types of fertilizer were based on the recommendations of^50^ and adapted by^51^ for the common bean crop. The experiment was conducted with 3 plants per pot, with each pot considered a treatment.

### Germplasm used

The germplasm used was collected from rural communities and street markets, belonging to the "Carioca" variety, with the following standard characteristics: grains with light cream color, brownish stripes, and no halo. Common bean cultivars with contrasting levels of efficiency were used in the organic fertilizer. Plant introduction obtained from the Federal University of Uberlândia (UFU) campus, located in the city of Monte Carmelo, Minas Gerais, Brazil, and freely distributed for research purposes. The common bean cultivars, with a response to the organic fertilizer, were obtained by D.J. Marques, one of the co-authors of this article. The experimental protocols involving plant materials and the analyses were conducted per the institutional and international guidelines of the creators of the methods.

To obtain UFU-1, hybridization was performed between the UFU-4 and UFU-7 cultivars following the methodology described by Peternelli and Borém (1999), resulting in a hybrid seed, which was sown together with the parents during the experiment.

### Soil preparation

The soil used for the research was a Dystrophic Red Latosol (Oxisol) with clay texture, collected in a native forest (18° 43′ 48.3″ S, 47° 30′ 16.6″ W). Soil samples were taken at a depth of 0–20 cm, air-dried, sieved, and homogenized for the determination of physical and chemical characteristics. The chemical analysis of the soil sample is shown in Table 1.

Soil analysis was also performed for the fertilization (organic × mineral fertilizer) after the plant cycle was completed. Individual samples were taken from each pot at a depth of 0 to 20 cm, and later combined to form a composite sample that was sent to the laboratory (Table 1).

Subsequently, based on the data obtained in the analysis, the fertilizer calculation was carried out, using commercial mineral fertilizer for mineral fertilizer and composting was performed based on bovine manure and vegetable waste collected in the area for organic fertilization. Composting was done in layers of grass followed by manure with mineral compounds, and this process was repeated several times to form a one-meter-high pile. The pile was left to decompose for a period of 4 months and regularly turned and watered as needed. A compost sample was later collected and subjected to analysis (Table 2). Subsequently, once the experiment was carried out, soil samples were taken from the containers and soil fertility analyzes were carried out, the result were compared with the initial soil before the experiment (Table 3).

**Table 2:** Analysis of compost used in organic fertilization.

Analyzes	Unit	Dry base 110 °C	Natural humidity
pH CaCl_2_ 0,01 M	pH	–	5.78
Density	g cm^3^	–	0.59
Moisture lost to 60–65 °C	%	–	6.90
Moisture lost to 65–110 °C	%	–	2.16
Total humidity	%	–	9.06
Inert materials	%	–	0.00
Total nitrogen	%	0.83	0.75
OM. total (combustion)	%	26.57	24.16
OM. compostable (titration)	%	25.82	23.48
OM. composting	%	0.75	0.68
Total carbon	%	14.76	13.42
Organic carbon	%	14.34	13.04
Total mineral residue	%	75.05	68.25
Insoluble mineral residue	%	53.52	48.67
Soluble mineral residue	%	21.54	19.58
Relationship C-total and N-total	–	18/1	18/1
Relationship C- organic e N-total	–	17/1	17/1
Phosphor (PO-total)	%	0.54	0.49
Potassium (KO-total)	%	0.83	0.75
Calcium (Ca-total)	%	0.80	0.73
Magnesium (Mg-total)	%	0.17	0.15
Sulfur (S-total)	%	0.07	0.06
Boron (B-total)	mg kg	27.00	25.00
Copper (Cu-total)	mg kg	50.00	45
Iron (Fe-total)	mg kg	47,402.00	43,106
Manganese (Mn-total)	mg kg	171.00	156
Zinc (Zn-total)	mg kg	56.00	51

**Table 3:** Chemical and physical analysis of soil fertility before planting (1), chemical analysis of organic fertilizer (2), mineral fertilizer (3) after harvesting the common bean, and physical analysis (4).

Elements	Unit	(1)	(2)	(3)
*Chemical analyse*				
pH (H_2_O)	1: 2.5	5.40	6.10	6.2
pH (CaCl_2_)	1: 2.5	4.80	5.70	5.6
P meh	mg dm^−3^	0.90	29.50	31.8
P remaining	mg dm^−3^	6.50	–	–
P residual	mg dm^−3^	12.00	–	–
P total	mg dm^−3^	253.00	–	–
K^+^	mg dm^−3^	97.00	88.00	68.00
S-SO_4_^2^	mg dm^−3^	9.00	8.00	35.00
K^+^	cmol_c_ dm^−3^	0.25	0.22	0.17
Ca^2+^	cmol_c_ dm^−3^	1.25	2.10	3.50
Mg^2+^	cmol_c_ dm^−3^	0.75	0.80	0.80
Al^3+^	cmol_c_ dm^−3^	0.09	0.00	0.00
H + Al	cmol_c_ dm^−3^	3.00	2.10	2.10
SB	cmol_c_ dm^−3^	2.25	3.08	4.49
T	cmol_c_ dm^−3^	2.34	3.08	4.49
t	cmol_c_ dm^−3^	5.25	5.18	6.59
V	%	43.00	60.00	68.00
M	%	4.00	0.00	0
M.O	dag kg^−1^	29.00	1.70	1.80
C.O	dag kg^−1^	1.70	1.00	1.00
B	mg dm^−3^	0.22	0.09	0.12
Cu	mg dm^−3^	1.70	2.90	3.30
Fe	mg dm^−3^	23.00	19.00	24.00
Mn	mg dm^−3^	1.90	10.20	11.50
Zn	mg dm^−3^	0.60	7.00	8.60
*Physical analysis (4)*				
Sand	g kg^−1^	255		
Silt	g kg^−1^	100		
Clay	g kg^−1^	645		

Calculation for acidity correction was carried out to neutralize Al^3+^ and increase Ca^2^ and Mg^2+^ levels. Dolomitic limestone (CaCO_3_ + MgCO_3_) was used for soil acidity correction, applied to the pots, and hydrated and sealed with plastic bags for incubation for 30 days prior to the experiment to promote corrective reactions in the soil.

### Root volume

Before the plant root system samples were introduced into the greenhouse for dehydration and obtaining dry matter, root length was measured. The roots were spread on a bench for a period of 24 h after washing, and the length was measured with the help of a measuring tape, obtaining the result in centimeters. Subsequently, volumetric analysis of the roots was performed, and the volume was calculated using a graduated cylinder (1000 mL), which was filled with a solution composed of 70% alcohol and 30% water up to half of its capacity (500 mL), where the roots were immersed, and the volume was determined by the displacement of the solution and measured in milliliters (ml)^53^.

### Dry matter of the root and plant

Once the plants reached maturity, the leaves were collected before they fell off, and then transported to a desiccator where they remained for a period of three days at a temperature of 75 °C, with forced and constant ventilation. Subsequently, once the pods were harvested, the stem of the plants was removed, and the samples were transported again to the desiccator, where they remained for a period and temperature as previously specified, and the material was weighed to obtain the dry matter weight expressed in grams. Once the plants completed their cycle, irrigation was stopped, the soil was washed with running water, and a 5 mm spaced sieve was used to pack the roots, thus exposing the plant's root system, which was transported to the oven under the previously described conditions, obtaining values of root dry matter, expressed in grams.

### Agronomic evaluations

The plant cycle was evaluated by the number of days to reach flowering (R1) and the number of days required to reach pod maturity (R9).

Plant height (cm) was measured using a measuring tape from the plant collar to the main stem.

After harvesting the leaves, data on the length and width of the central leaflet were collected, the average was calculated, and subsequently the calculation of the leaf area index was performed.

After the pods were opened, information on seed height, length, and thickness was collected using a caliper, and then the weight of 100 seeds was measured, with the results expressed in grams, and finally, seed moisture was calculated based on weight.

Plant productivity (g plant^−1^) and grain yield estimated (Mg ha^−1^) were estimated based on the total number of pods per plant, the average number of seeds per pod, the weight of 100 seeds, and seed moisture, with the averages calculated in g/plot. For comparative purposes, productivity was estimated considering a population of 250.000 plants per hectare, and the results were transformed into g plant for Mg ha^−1^.

### Leaf area index

The leaf area index was estimated (R1) after collecting leaf samples by measuring their length and width. Due to the oval shape of the leaves, 30% of the found value was subtracted, and they were then individually weighed to obtain the specific leaf area index, expressed in square centimeters per gram (cm^2^/g), and transformed into square meters per kilogram (m^2^/kg). Subsequently, with the collection of all the plant leaves, the total dry matter weight of the leaves was weighed, and the leaf area index was estimated using the equation^54^.

### Reflectance index

For the SPAD index determination, a portable chlorophyll meter, SPAD-502, which provides instant non-destructive readings, was used to take two readings of the central leaflet of the plants, with the first reading taken at the upper third and the second in the middle third of the plants. Following the device's specifications, 3 readings were taken per central leaflet of a plant, and readings were taken for 3 central leaflets for each treatment, considering the average for tabulating the data. The measurements were made on October 20, 2022, at 8 am.

#### Pigment content in leaves

For the determination of the chlorophyll *a*, chlorophyll *b*, total chlorophyll, and carotenoid pigment content, leaves of the fourth trifoliate (without petiole), from the apex, on the main stem at the beginning of flowering (R1 stage) were collected. The leaves were crushed, and a mass of 0.5 g was added to a solution of petroleum ether and acetone (1:1) for a period of 24 h in the dark at 4 °C. Subsequently, absorbance was measured in a UV-190 spectrophotometer at wavelengths of 645, 652, 663, and 470 nm. The pigments present in the leaves were calculated based on absorbance values (mg 100 g^−1^ of fresh tissue) following the procedure described by^55^ and^56^.

#### Statistical analysis

The results were subjected to analysis of variance (Scott-Knott or t-test) according to the method proposed by^57^. In addition, standard deviations were calculated, and regression and correlation estimators were applied when relevant (Pearson or Spearman) using SAS software^58^.

Additionally, the data were subjected to a multivariate statistical approach, in which the dissimilarity between treatments was estimated. The dissimilarity matrix was obtained by the generalized distance of Mahalanobis (D2) as a measure of dissimilarity. The dissimilarity was represented by a dendrogram obtained by the Unweighted Pair Group Method using Arithmetic averages—UPGMA. The cutoff point was established at the location where an abrupt change was observed in the branches present in the dendrogram^14^.

The hierarchical clustering was validated by the cophenetic correlation coefficient, which was tested for significance by means of a t-test at a 5% probability level. The relative contributions of each variable to the dissimilarity (%) were estimated by the proposed method^15^.

## Data Availability

The datasets generated and/or analyzed during the current study are not publicly available due to the industrial safety of the cultivars that will be registered but are available from the corresponding author upon reasonable request.
